# Point‐of‐care Lung ultrasound assessment of positional changes in COVID‐19 ARDS in intensive care: A case report and review of the literature

**DOI:** 10.14814/phy2.70484

**Published:** 2025-08-22

**Authors:** Oguzhan Koca, Guniz Koksal

**Affiliations:** ^1^ Department of Immunology and Inflammation, Faculty of Medicine Imperial College London London UK; ^2^ Department of Internal Medicine, Cerrahpasa Faculty of Medicine Istanbul University‐Cerrahpasa Istanbul Turkey; ^3^ Division of Intensive and Critical Care, Department of Internal Medicine, Cerrahpasa Faculty of Medicine Istanbul University‐Cerrahpasa Istanbul Turkey

**Keywords:** ARDS, case report, COVID‐19, lung ultrasound, positioning

## Abstract

Prone positioning is a cornerstone in the management of acute respiratory distress syndrome (ARDS), including COVID‐19‐related ARDS. However, alternative positioning strategies such as reverse Trendelenburg position (RTP) have received limited attention. The objective is to evaluate the physiological effects of RTP on lung aeration using lung ultrasound (LUS) in a patient with severe COVID‐19 ARDS. We performed serial LUS assessments across 12 lung regions in a mechanically ventilated ARDS patient undergoing three different positional changes: Trendelenburg, flat supine/prone, and reverse Trendelenburg. LUS scores were used to quantify regional aeration and global lung aeration. Oxygenation parameters were monitored in parallel. RTP was associated with a reduction in global LUS score (from 21 to 17), indicating improved lung aeration, particularly in the superior and posterior regions. Oxygenation improved concurrently, with increases in peripheral and central venous oxygen saturations. No significant change in lung compliance was observed. This case demonstrates that RTP may facilitate redistribution of extravascular lung water and improve aeration in select lung regions. LUS offers a dynamic, noninvasive method to assess and guide individualized positioning strategies in ARDS. These findings support further exploration of vertical positioning as an adjunct in ARDS management when prone positioning is not feasible.

## Graphical abstract

Point‐of‐care lung ultrasound (LUS) was used to assess changes in lung aeration across Trendelenburg, flat, and reverse Trendelenburg positions in a mechanically ventilated patient with COVID‐19 ARDS. Reverse Trendelenburg improved superior lung aeration and oxygenation without altering compliance, as shown by the LUS‐based aeration gradient.

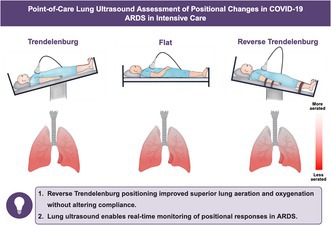

## 1 INTRODUCTION

Acute respiratory distress syndrome (ARDS) is a life‐threatening condition characterized by severe hypoxemia and decreased lung compliance, often requiring mechanical ventilation and advanced supportive strategies. The emergence of coronavirus disease 2019 (COVID‐19) has significantly increased the incidence of ARDS, with reports suggesting that approximately 15%–30% of hospitalized COVID‐19 patients develop ARDS (Attaway et al., 2021). Managing ARDS involves a combination of lung‐protective ventilation strategies, prone positioning, and neuromuscular blockade to optimize oxygenation and reduce ventilator‐induced lung injury (Acute Respiratory Distress Syndrome Network et al., 2000; Guérin et al., 2018; Papazian et al., 2010; Tonelli et al., 2014). Among these, prone positioning has been demonstrated to improve survival, particularly in severe ARDS cases (Charron et al., 2011; Gattinoni et al., 2010; Guérin et al., 2013; Hu et al., 2014; Sud et al., 2010).

However, the implementation of prone positioning is often challenging due to contraindications such as hemodynamic instability, pressure ulcers, and airway management difficulties (Grasselli et al., 2023; Guérin et al., 2020; Kuljit et al., 2021). As a result, alternative positioning strategies, including Trendelenburg and reverse Trendelenburg positions, have been explored to assess their impact on lung mechanics and oxygenation. Some studies suggest that Trendelenburg positioning may improve lung compliance without significantly enhancing oxygenation (Kodamanchili et al., 2022; Marrazzo et al., 2022; Rezoagli et al., 2021). Conversely, vertical positioning strategies, including reverse Trendelenburg, have been associated with improved oxygenation by reducing abdominal compression and facilitating lung recruitment (Dellamonica et al., 2013; Mynster et al., 1996; Richard et al., 2006). Despite these findings, there is limited data on the ultrasonographic evaluation of lung aeration changes across different positioning strategies in ARDS patients, particularly in the context of COVID‐19.

Lung ultrasound (LUS) has emerged as a valuable bedside tool for monitoring lung aeration and guiding clinical decisions in ARDS management (Bouhemad et al., 2007, 2015; Mongodi et al., 2021). LUS has been used to quantify lung aeration loss, predict response to recruitment maneuvers, and assess the effectiveness of positioning strategies (Bouhemad et al., 2011; Kim et al., 2020; Wu et al., 2022). While studies have demonstrated the utility of LUS in monitoring lung aeration changes during prone positioning (Haddam et al., 2016; Prat et al., 2016; Wang et al., 2016), there is a paucity of data on its role in evaluating alternative positioning strategies such as reverse Trendelenburg. Given the dynamic nature of lung aeration in ARDS, a better understanding of how positioning impacts LUS findings could help optimize ventilation strategies and improve patient outcomes.

In this study, we present the use of LUS to monitor lung aeration changes in a COVID‐19 ARDS patient undergoing different positioning maneuvers, along with a comprehensive review of the literature. This case report was prepared in accordance with the CARE guidelines, and the completed CARE checklist is provided as Appendix S1. Our findings highlight the potential benefits of reverse Trendelenburg positioning in improving oxygenation and reducing lung aeration loss, providing new insights into individualized positioning strategies for ARDS patients.

## 2 CLINICAL APPLICATION: A CASE REPORT

A 73‐year‐old male patient presented to an external medical center with complaints of fever and cough. Thoracic computed tomography revealed pneumonic consolidations, prompting hospital admission. The patient was empirically started on piperacillin‐tazobactam, levofloxacin, molnupiravir, and oseltamivir due to pneumonia based on CT findings. However, his oxygen requirement increased within 24 h, leading to his transfer to our intensive care unit with type 1 respiratory failure. A polymerase chain reaction (PCR) test confirmed COVID‐19 infection.

Four days after ICU admission, the patient was orotracheally intubated and diagnosed with COVID‐19‐related ARDS. Standard ARDS management was initiated, including lung‐protective ventilation with low tidal volume, prone positioning, and neuromuscular blockade. Despite optimal sedation and muscle paralysis, oxygenation remained inadequate. To further assess lung aeration and positional effects on pulmonary mechanics, a bedside LUS was performed. LUS revealed significant aeration loss, particularly in the superior lung zones. Consequently, the patient was repositioned to reverse Trendelenburg (20° head elevation) under sedation and paralysis to enhance aeration in the superior lung regions.

Following the transition to reverse Trendelenburg, oxygenation parameters improved, with SpO_2_ increasing from 85% to 88% and ScvO_2_ from 68% to 79.5%, corresponding to an increase in ScvO_2_/FiO_2_ ratio from 113 to 132.5, without a change in lung compliance. On the 24th day of ICU admission, the patient developed gram‐negative septicemia, multi‐organ dysfunction syndrome (MODS), decreased urine output, and increased extravascular fluid volume. Continuous renal replacement therapy (CRRT) with an oXiris filter was initiated. All LUS assessments and physiological measurements were performed prior to the onset of MODS and the initiation of CRRT, thereby excluding potential confounding effects from evolving systemic deterioration. Despite these interventions, the patient's condition deteriorated, and he passed away due to MODS on the 30th day of hospitalization.

## 3 LUNG ULTRASOUND

**FIGURE 1:**
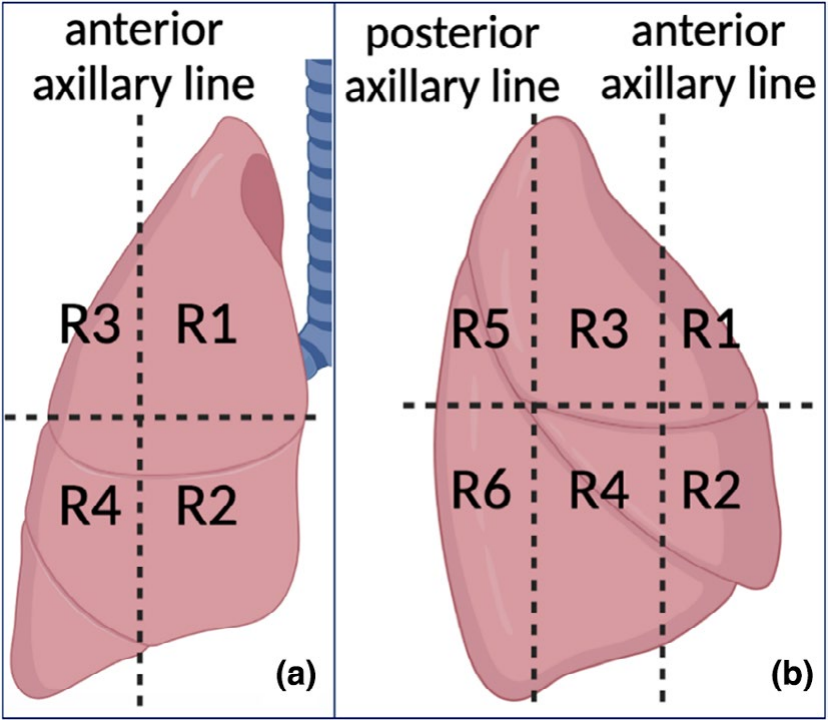
Regions for standardized lung ultrasound examination. For demonstration, the right lung is divided into anterior, lateral, and posterior regions based on anatomical landmarks (anterior and posterior axillary lines and internipple line). (a) Anterior view of the lung. (b) Lateral view of the lung. The regions are defined as follows: R1: Region 1, anterior superior; R2: Region 2, anterior inferior; R3: Region 3, lateral superior; R4: Region 4, lateral inferior; R5: Region 5, posterior superior; R6: Region 6, posterior inferior. Created in BioRender.

Point‐of‐care LUS was performed using the six‐region protocol per hemithorax: anterosuperior (1), anteroinferior (2), laterosuperior (3), lateroinferior (4), posterosuperior (5), and posteroinferior (6) (Figure 1) (Bouhemad et al., 2015; Mongodi et al., 2021). A Mindray DC‐80 system was used. Aeration loss was scored using the LUS score: normal aeration (no more than two B‐lines) = 0; moderate loss of aeration (three or more well‐separated B‐lines) = 1; severe loss of aeration (coalescent B‐lines, white lung) = 2; complete loss of aeration (tissue‐like pattern or consolidation) = 3 (Bouhemad et al., 2015). The global LUS score was the sum of 12 regional scores.

The patient was examined in the supine Trendelenburg position (−20^°^), supine flat position, and supine reverse Trendelenburg position (20^°^) respectively, for anterior and lateral regions (Region 1–4). The examination was repeated in the prone Trendelenburg position, prone flat position, and reverse Trendelenburg position respectively, for posterior regions (Region 5–6). As the patient had severe ARDS, neuromuscular blockade and prone positioning were performed according to our routine institutional positioning procedure. Ultrasound was performed after routine position changes.

**FIGURE 2:**
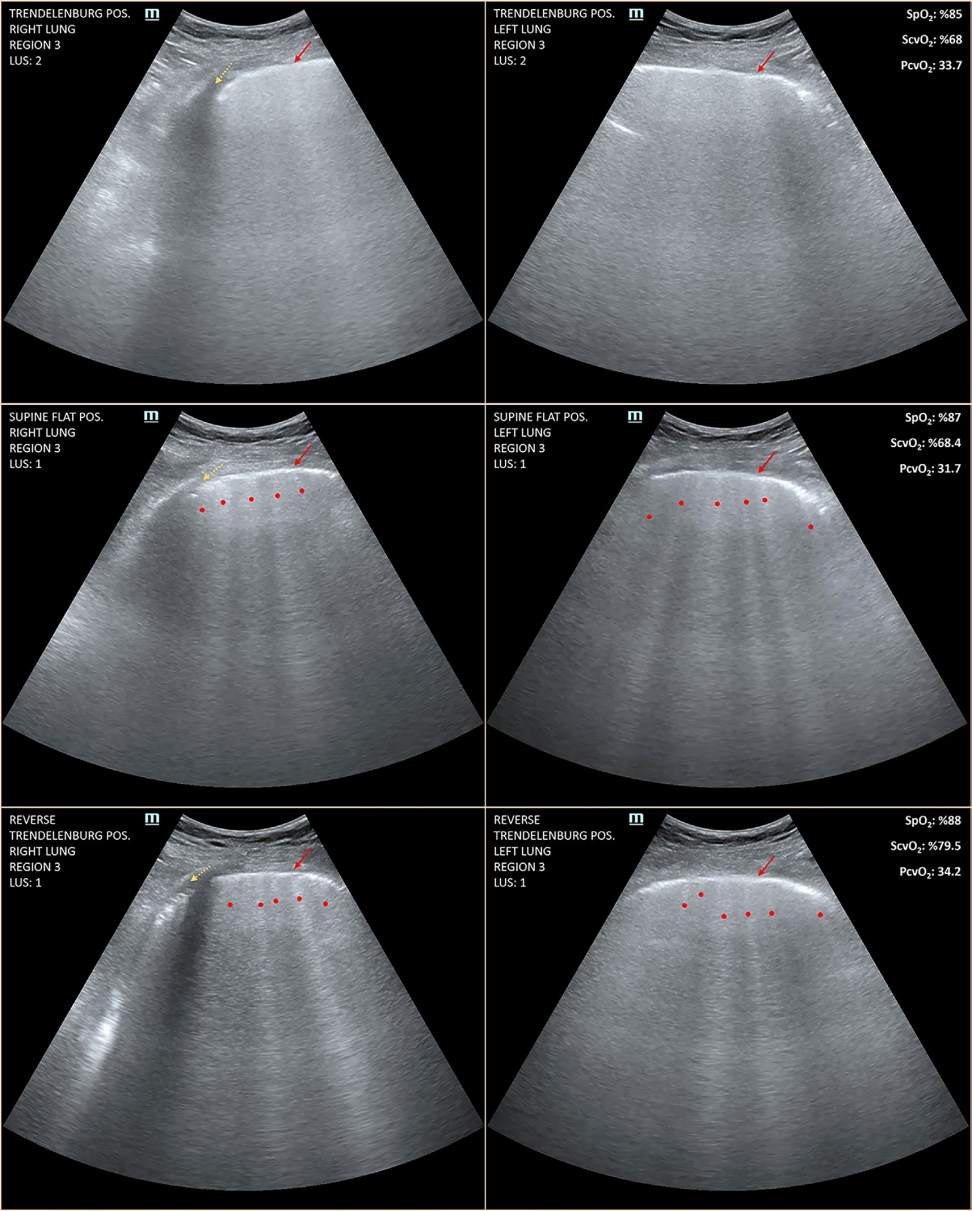
Ultrasonographic findings corresponding to loss of aeration with the position. At region 3, LUS score was 2 (white lung) for both lungs in the Trendelenburg position; 1 (well‐separated B‐lines) for both lungs in the supine flat position; and 1 (well‐separated B‐lines) for both lungs in the reverse Trendelenburg position. The red arrow indicates pleural line; the yellow arrow indicates pleural effusion; the red dots indicate for B‐lines. LUS score, lung ultrasound score; PcvO_2_, central venous O_2_ pressure; ScvO_2_, central venous O_2_ saturation; SpO_2_, peripheral O_2_ saturation. Consent for publication of the ultrasound images was obtained from the patient's legal representative.

Global LUS scores were 21, 18, and 17 in the Trendelenburg, flat, and reverse Trendelenburg positions, respectively. A four‐point difference in the global LUS score was observed between the Trendelenburg and reverse Trendelenburg positions (21 vs. 17). The main difference according to the positions was observed in the laterosuperior and posterosuperior regions (regions 3 and 5); at region 3, the LUS score was 2 for both lungs in the Trendelenburg position, but 1 for both lungs in the reverse Trendelenburg position, and at region 5, the LUS score was 3 and 2 for the right and left lungs in the Trendelenburg position, but 1 for both lungs in the reverse Trendelenburg position (Figure 2). A full set of lung ultrasound images covering all 36 regions is provided in Appendix S2. Ventilator parameters and the duration maintained in each position prior to LUS assessment are summarized in Table S1.

## 4 DISCUSSION

COVID‐19 has caused significant global morbidity and mortality, with 15%–30% of hospitalized patients developing ARDS (Attaway et al., 2021). While clinical, ventilatory, and laboratory parameters are essential in ARDS management, radiological imaging remains critical for monitoring disease progression. However, imaging modalities may pose logistical and infection control challenges, especially in critically ill or contagious patients.

Challenges associated with cross‐sectional imaging in ARDS include infection control concerns, prolonged equipment downtime for disinfection, and the inability to transport critically ill patients safely. As a result, bedside ultrasonography has gained widespread adoption during the COVID‐19 pandemic due to its portability, ease of sterilization, and real‐time applicability at the patient's bedside.

LUS has emerged as an ideal tool for dynamically monitoring aeration loss in ARDS, particularly in COVID‐19 patients (Lichter et al., 2020; Zieleskiewicz et al., 2020). Beyond diagnostic use, several studies have demonstrated the utility of LUS scores in evaluating therapeutic responses, such as recruitment maneuvers (Bouhemad et al., 2011; Kim et al., 2020; Wu et al., 2022). Notably, LUS has been shown to correlate with pressure–volume curves in assessing PEEP‐induced recruitment (Bouhemad et al., 2011), and ultrasound‐guided strategies may outperform traditional sustained inflation methods in optimizing perioperative respiratory function (Wu et al., 2022).

Among various interventions, low tidal volume ventilation, neuromuscular blockade, and prone positioning (PP) have been shown to improve survival in ARDS (Acute Respiratory Distress Syndrome Network et al., 2000; Charron et al., 2011; Gattinoni et al., 2010; Guérin et al., 2013, 2018; Hu et al., 2014; Papazian et al., 2010; Sud et al., 2010; Tonelli et al., 2014). PP is recommended in patients with a PaO_2_/FiO_2_ ratio < 150 mmHg (Grasselli et al., 2023; Guérin et al., 2013, 2020). Despite strong evidence, its application remains limited; the APRONET study reported PP use in only 32.9% of severe ARDS cases (Guérin et al., 2018). These recommendations also apply to COVID‐19–associated ARDS (Grasselli et al., 2023; Kuljit et al., 2021; World Health Organization, 2020).

Unlike prone positioning, alternative techniques such as Trendelenburg or reverse Trendelenburg lack high‐level evidence. Observational studies have reported improved lung compliance with Trendelenburg positioning or chest wall compression, likely due to reduced tidal overinflation (Kodamanchili et al., 2022; Rezoagli et al., 2021). However, these interventions have not consistently improved oxygenation. A recent study also showed increased compliance, but no oxygenation benefit, when COVID‐19 patients were moved from a semi‐recumbent to a flat supine position (Marrazzo et al., 2022). These findings suggest that improved mechanics do not always translate into gas exchange improvement and highlight the complexity of positioning strategies in ARDS.

In contrast, several studies have reported physiological benefits of trunk verticalization in ARDS patients (Dellamonica et al., 2013; Richard et al., 2006). A French observational study comparing supine and semi‐upright (45° trunk, 45° legs‐down) positions in 16 patients demonstrated significant improvement in oxygenation and lung volume in the vertical posture (Richard et al., 2006). The authors attributed this to reduced abdominal compression, enhanced diaphragmatic excursion, and recruitment of dependent lung zones. As compliance did not increase, the observed lung volume gain likely reflected true recruitment rather than mechanical improvement (Richard et al., 2006). Building on these findings, subsequent multicenter studies have explored how different vertical positions affect oxygenation and lung volume in ARDS. A multicenter study in 40 ARDS patients assessed oxygenation and end‐expiratory lung volume (EELV) across various upright positions, including semi‐recumbent and seated postures (Dellamonica et al., 2013). Verticalization improved oxygenation in 32% of patients, with corresponding increases in EELV (Dellamonica et al., 2013). Notably, responders—those with >20% PaO_2_/FiO_2_ improvement—had lower compliance, suggesting that lung recruitment rather than mechanics underlies the observed benefit, consistent with prior findings (Dellamonica et al., 2013; Richard et al., 2006).

However, not all upright positions yield respiratory benefit. One study found no oxygenation improvement in the semi‐recumbent position, likely due to maintained abdominal compression from the elevated trunk and horizontal legs (Bittner et al., 1996). Supporting this, semi‐recumbent posture has been shown to increase intra‐abdominal pressure by approximately 9 mmHg (De Keulenaer et al., 2009).

In our patient, vertical positioning improved ScvO_2_/FiO_2_ and SpO_2_ without changes in compliance, alongside a reduction in LUS scores. Unlike prior studies, this report provides ultrasonographic evidence of aeration improvement with verticalization. These findings support the hypothesis that gravitational redistribution contributes to recruitment in superior lung regions. Persistent extravascular lung water, despite fluid removal, may reflect altered capillary permeability related to sepsis.

The physiological benefit observed with reverse Trendelenburg positioning may be multifactorial. Vertical orientation of the trunk is known to reduce abdominal pressure, thereby enhancing diaphragmatic excursion and improving the distribution of transpulmonary pressure (Richard et al., 2006). This postural adjustment can facilitate alveolar recruitment in the dependent lung regions without necessarily increasing static compliance, as noted in our case. Moreover, by reducing gravitational compression on the posterior thorax and redistributing extravascular lung water, reverse Trendelenburg may optimize the ventilation‐perfusion ratio (V/Q) in dorsal lung zones, which are often compromised in ARDS. Taken together, the interplay between gravitational forces, thoracoabdominal mechanics, and hemodynamics likely underpins the observed physiological response, even in the absence of changes in compliance.

Compared to prone positioning, which redistributes perfusion and promotes dorsal recruitment through direct compression, reverse Trendelenburg primarily enhances aeration by reducing abdominal pressure and gravitational fluid shift without imposing the hemodynamic compromise often associated with prone posture. Moreover, reverse Trendelenburg positioning facilitates LUS acquisition by allowing unrestricted anterior and lateral chest access, thereby minimizing technical limitations often encountered in prone imaging. These advantages may support its use as an adjunct or alternative in patients who cannot tolerate prone positioning or require frequent physiological reassessment.

LUS score improvements suggest that gravitational redistribution of lung fluids influences aeration in ARDS. Superior lung regions (regions 3 and 5), aligned with the “baby lung” concept, demonstrated better aeration in the reverse Trendelenburg position. This may reflect reduced fluid loading and enhanced alveolar recruitment in these zones. Conversely, Trendelenburg positioning may worsen collapse by shifting extravascular fluid upward. These findings highlight the utility of dynamic LUS in tailoring physiologically informed positioning strategies.

Given the reduced functional residual capacity (FRC) in ARDS, vertical positioning may act as a physiological recruitment maneuver by increasing lung volume and improving oxygenation (Chiumello et al., 2008; Dellamonica et al., 2011). Reverse Trendelenburg or similar strategies could serve as adjuncts to prone positioning, particularly when prone therapy is interrupted due to complications such as pressure injuries or hemodynamic instability. Additionally, in patients requiring CRRT, the use of oXiris hemofilters may also contribute to improved oxygenation by adsorbing circulating inflammatory cytokines and reducing alveolar and interstitial edema, although this occurred after the ultrasound measurements in our case.

Although LUS has been widely studied in ARDS, its use in evaluating positional responses has primarily focused on prone positioning (Haddam et al., 2016; Ibarra‐Estrada et al., 2020; Prat et al., 2016; Wang et al., 2016). While some studies attempted to predict oxygenation response using LUS patterns, findings remain mixed (Haddam et al., 2016; Prat et al., 2016; Wang et al., 2016). In COVID‐19 patients on high‐flow nasal cannula, awake prone positioning reduced LUS scores in those who avoided intubation, suggesting prognostic utility (Ibarra‐Estrada et al., 2020). However, no prior reports have compared LUS findings across multiple positioning strategies beyond prone versus supine. This case provides the first LUS‐based evidence demonstrating differential aeration patterns with Trendelenburg and reverse Trendelenburg positioning in ARDS.

## 5 CONCLUSION

Vertical positioning may influence lung aeration patterns through gravitational mechanisms. In our case, reverse Trendelenburg improved oxygenation and aeration in superior regions, suggesting fluid redistribution and alveolar recruitment. Point‐of‐care lung ultrasound enabled real‐time visualization of these changes, supporting its role in physiology‐guided ventilation. When prone positioning is not feasible, vertical positioning may offer a practical, physiologically grounded alternative.

## FUNDING INFORMATION

This research did not receive any specific grant from funding agencies in the public, commercial, or not‐for‐profit sectors.

## CONFLICT OF INTEREST STATEMENT

The authors have no conflicts of interest to declare.

## ETHICS STATEMENT

This study was conducted following the ethical standards outlined in the World Medical Association Declaration of Helsinki (2013). Written informed consent was obtained from the patient's legally authorized representative.

## Supporting information


Appendix S1.

Appendix S2.

Table S1.

## Data Availability

Data will be made available on request.
