# Supplementary material for: Point‐of‐care Lung ultrasound assessment of positional changes in COVID‐19 ARDS in intensive care: A case report and review of the literature
Source: Physiol Rep. 2025 Aug 22;13(16):e70484. doi: 10.14814/phy2.70484 (PMC12371258; doi:10.14814/phy2.70484)
Supplement: Supplementary file 2 — Appendix S2. [file PHY2-13-e70484-s001.docx]

**Supplementary File 2. Full set of lung ultrasound images covering all 36 regions**

**TRENDELENBURG POSITION**

| **REGION** | **RIGHT LUNG** | **LEFT LUNG** |
| --- | --- | --- |
| **1** | 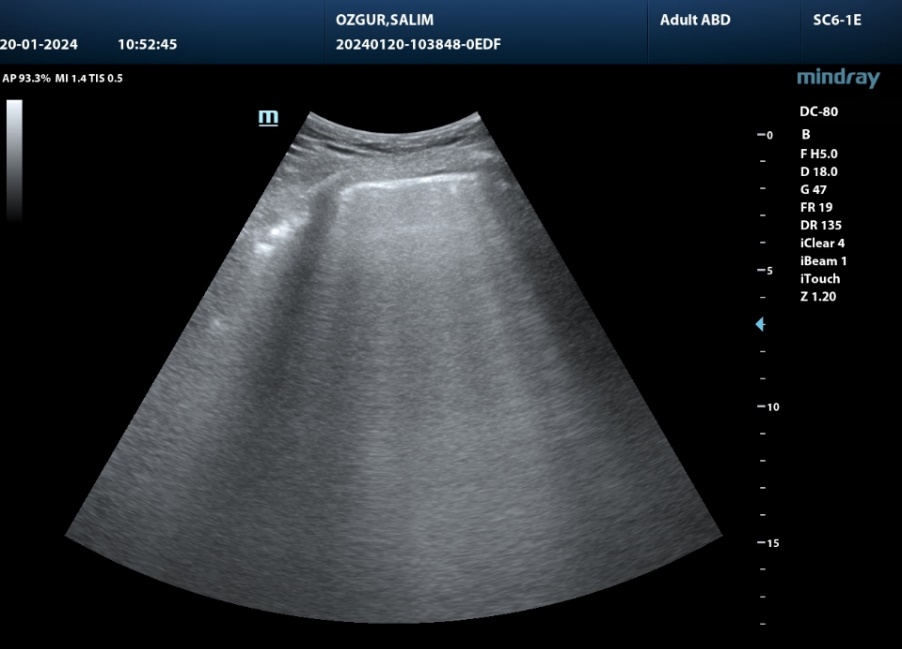 | 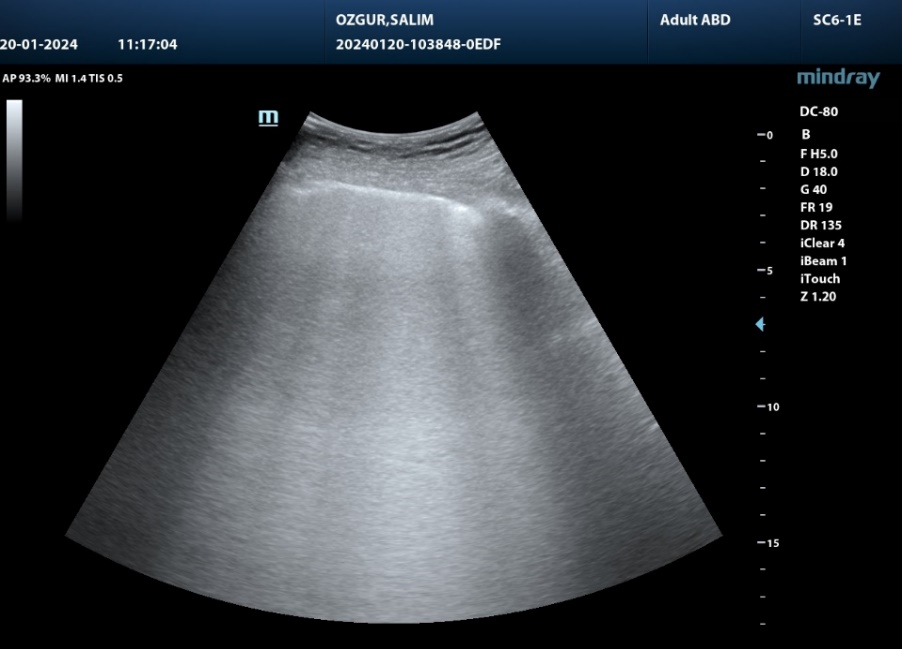 |
| **2** | 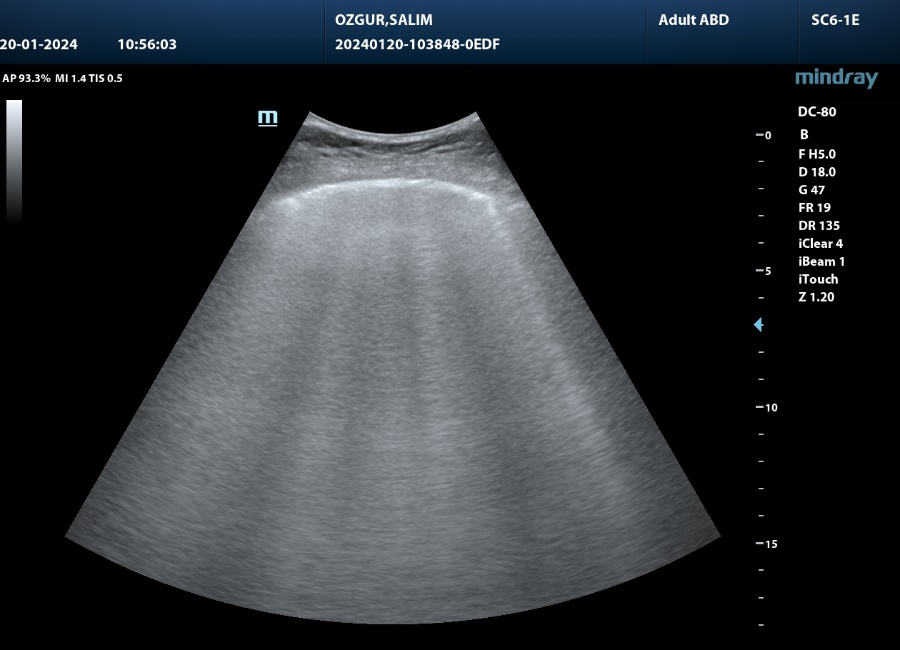 | 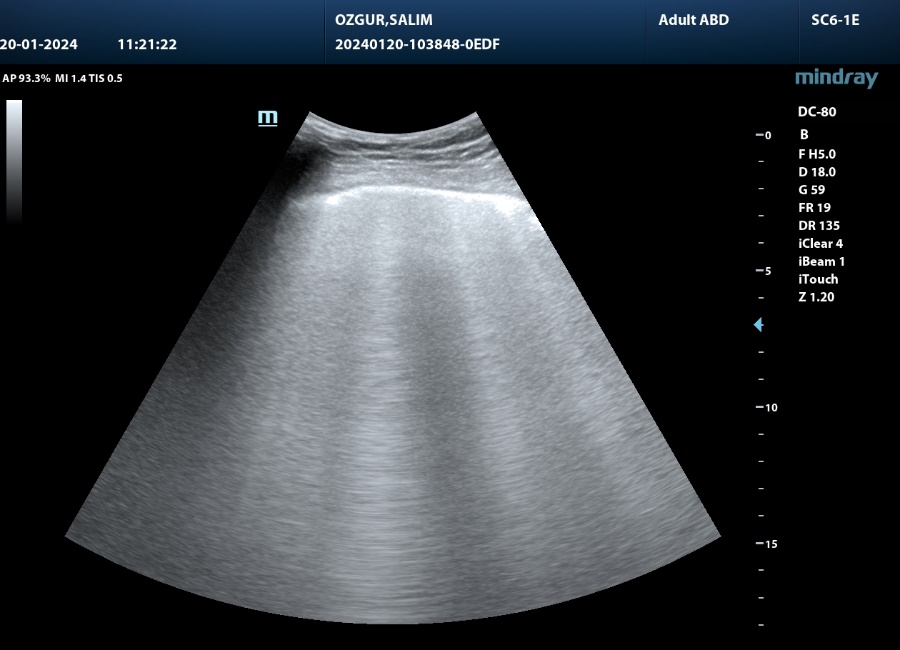 |
| **3** | 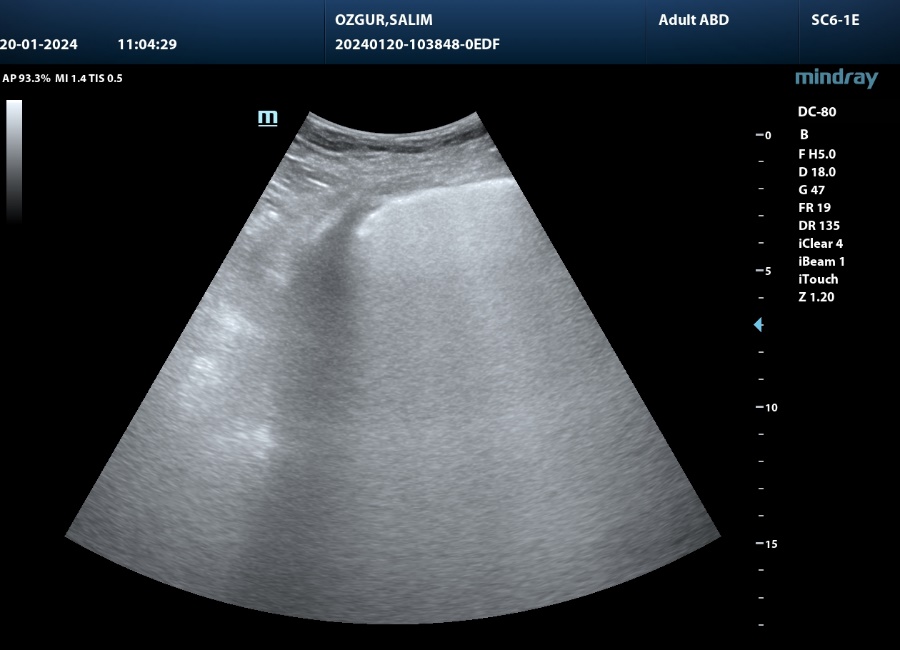 | 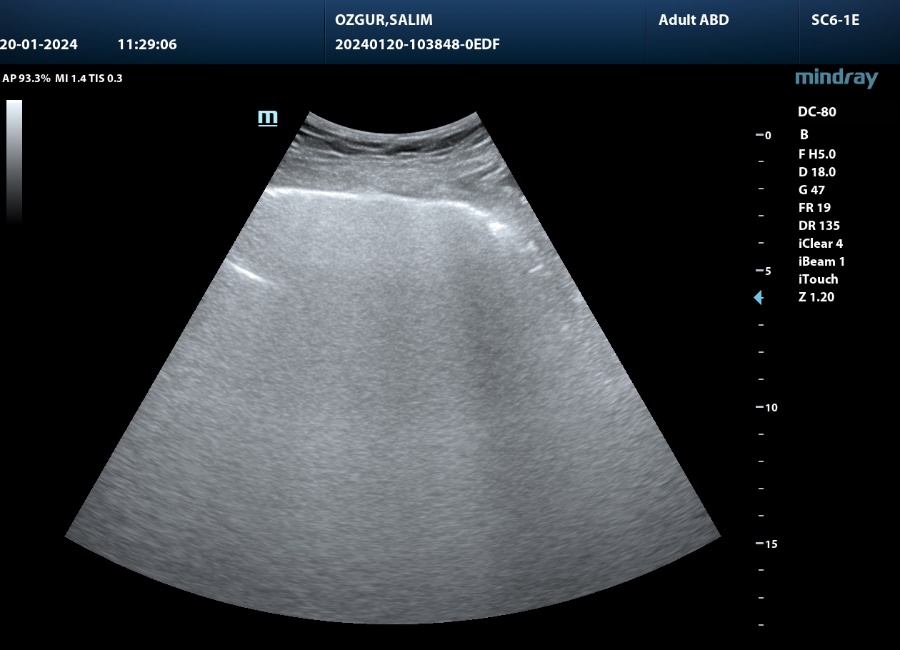 |
| **4** | 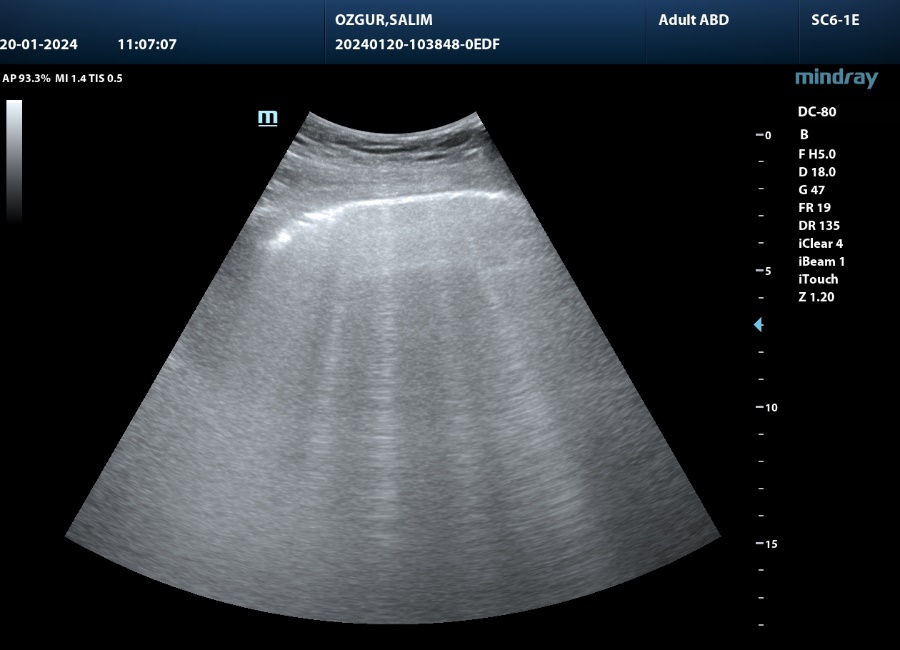 | 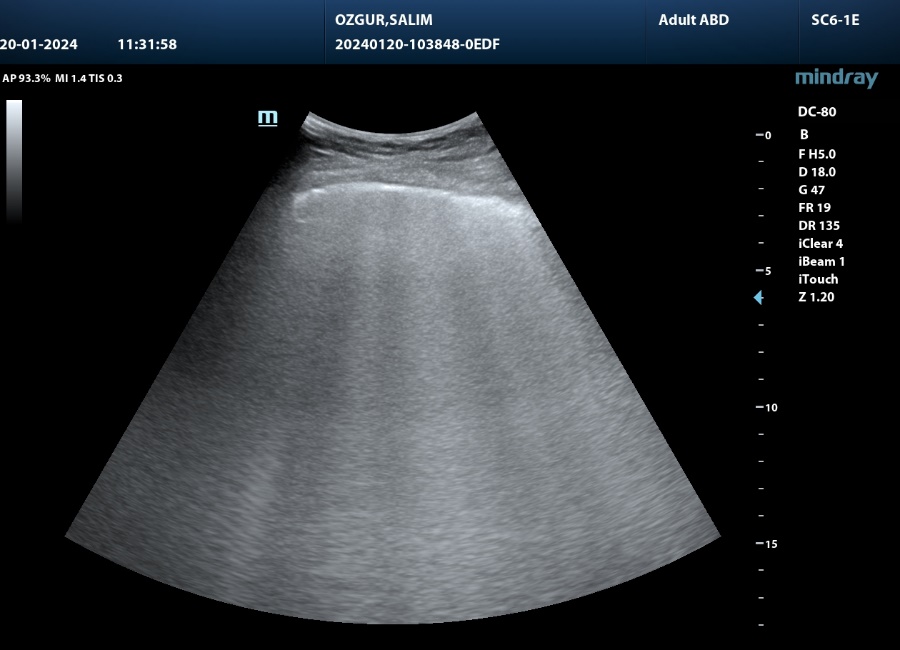 |
| **5** | 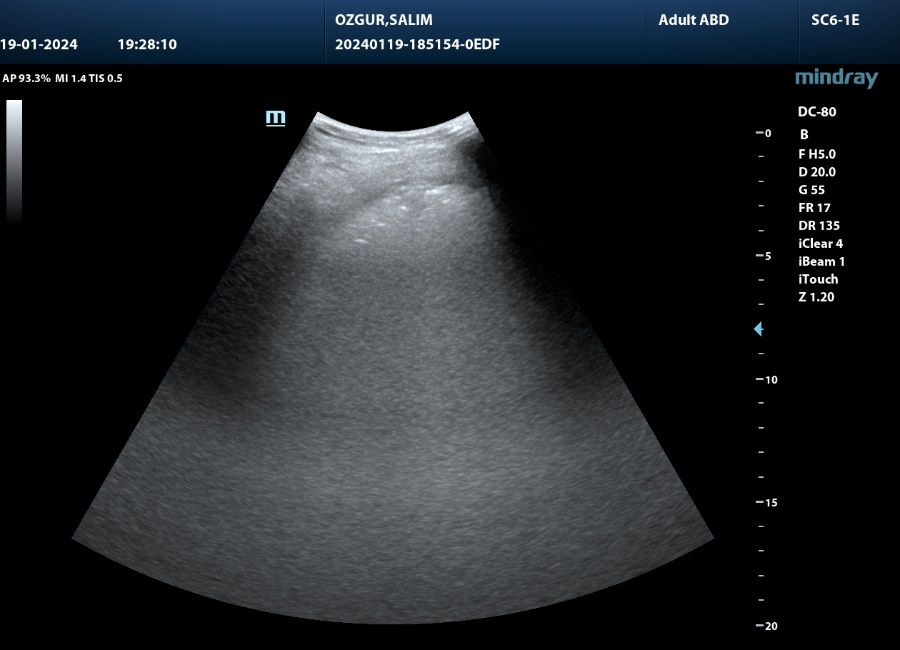 | 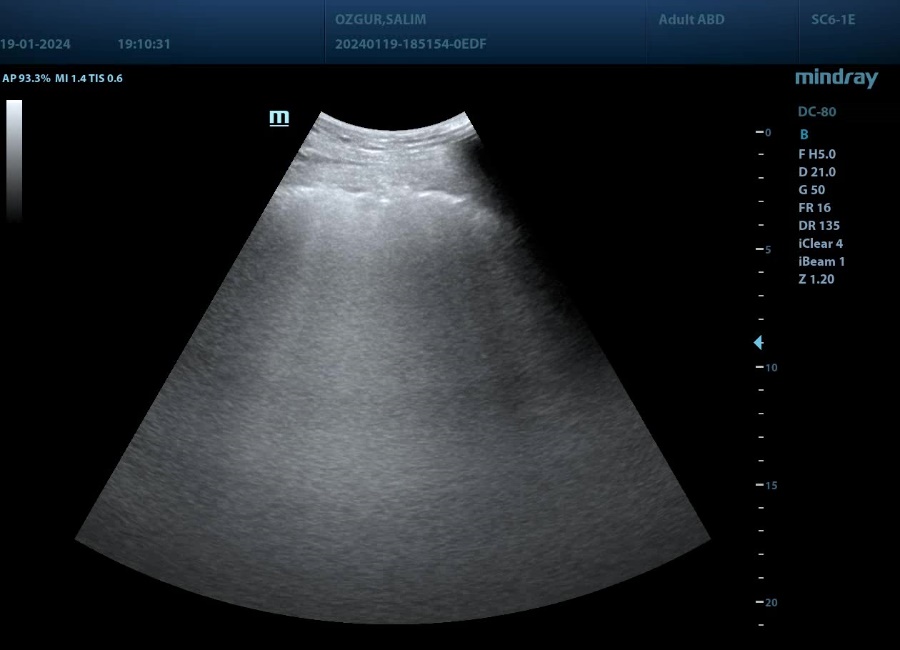 |
| **6** | 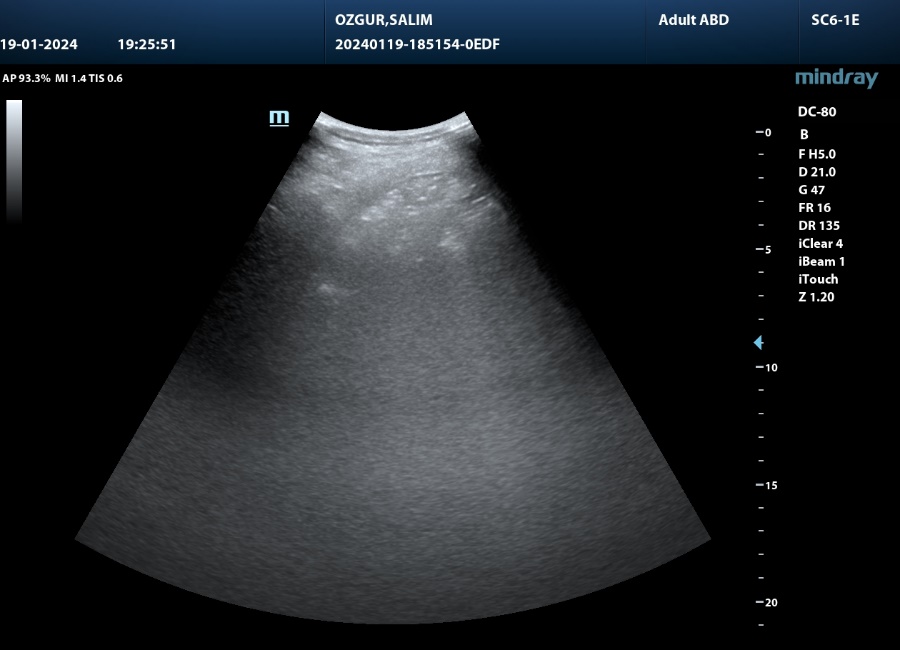 | 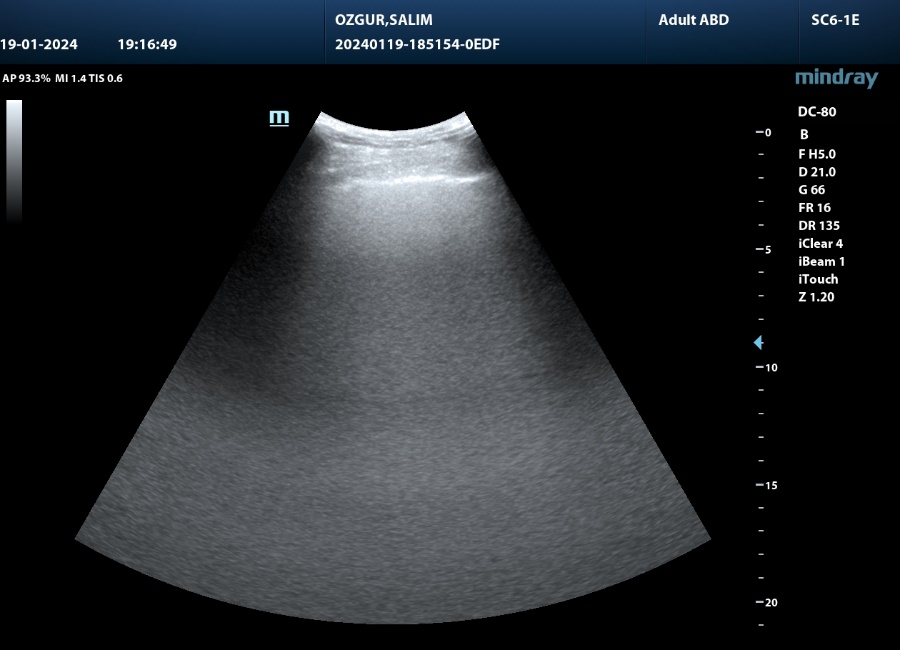 |

**0^0^ POSITION**

| **REGION** | **RIGHT LUNG** | **LEFT LUNG** |
| --- | --- | --- |
| **1** | 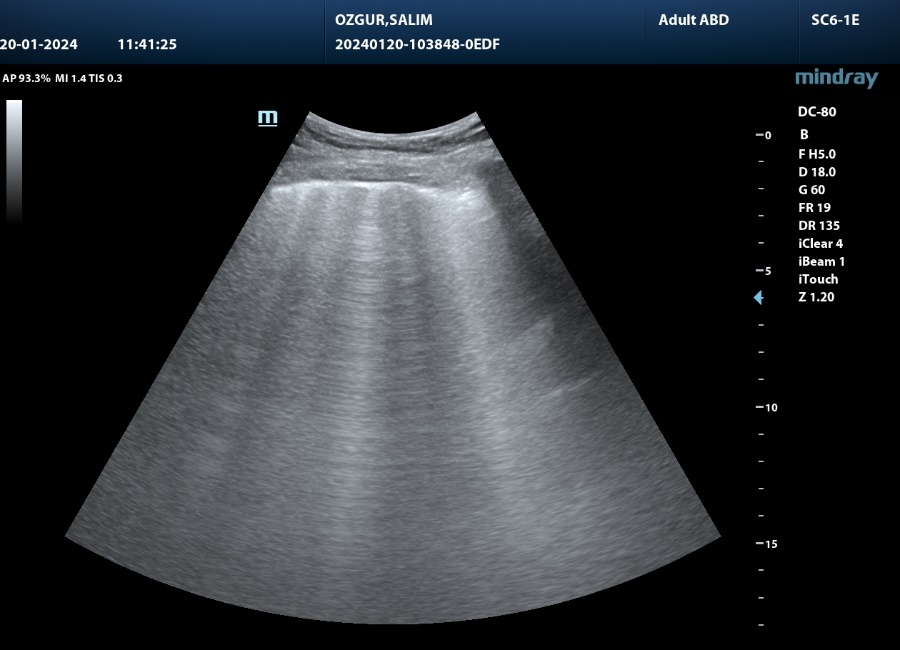 | 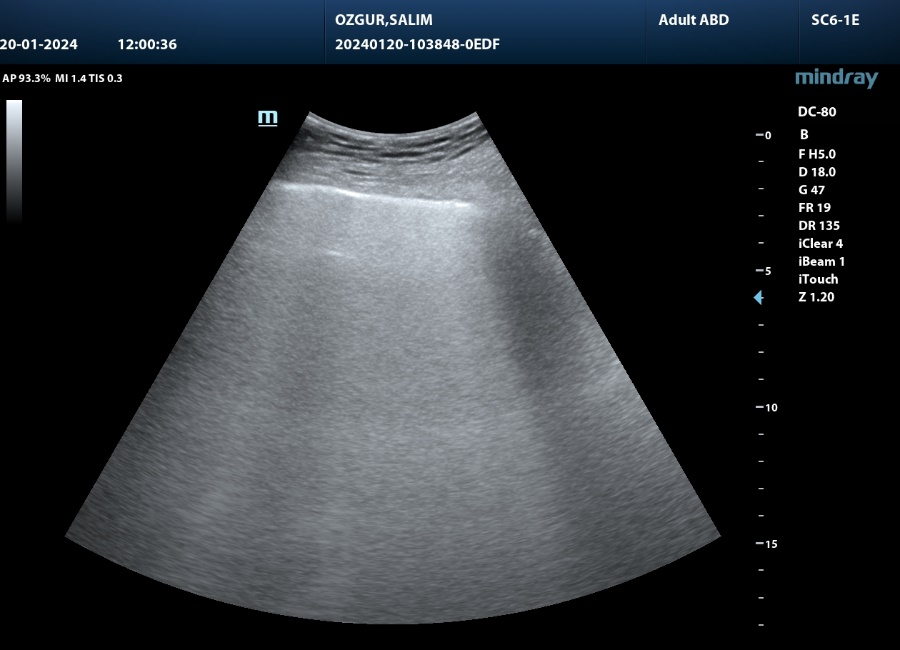 |
| **2** | 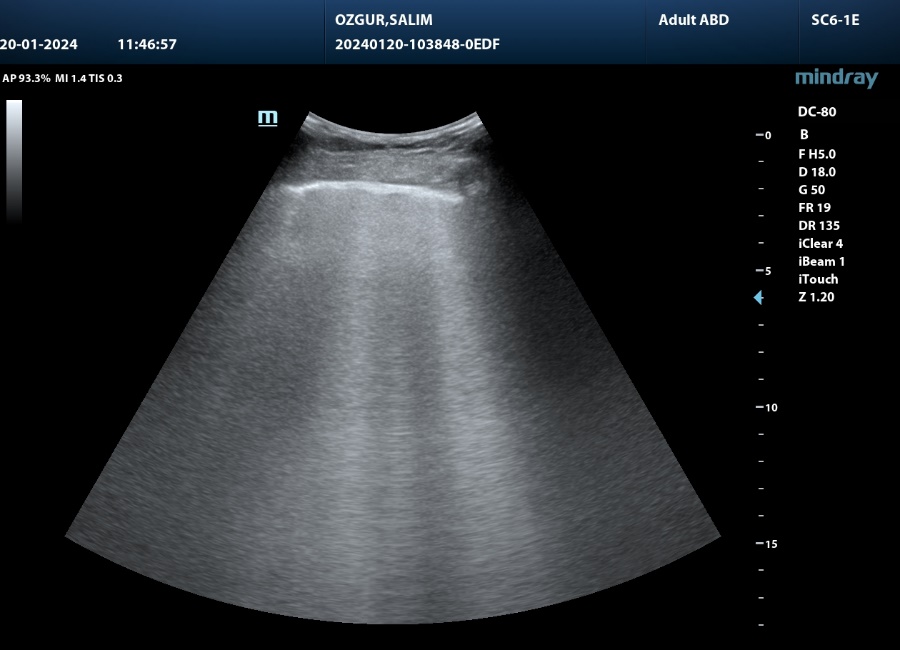 | 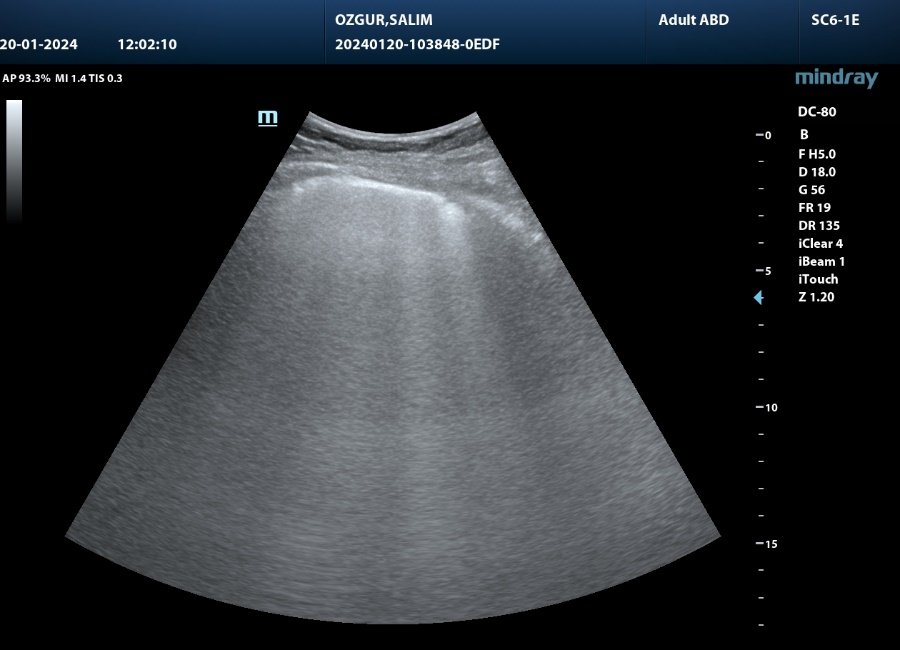 |
| **3** | 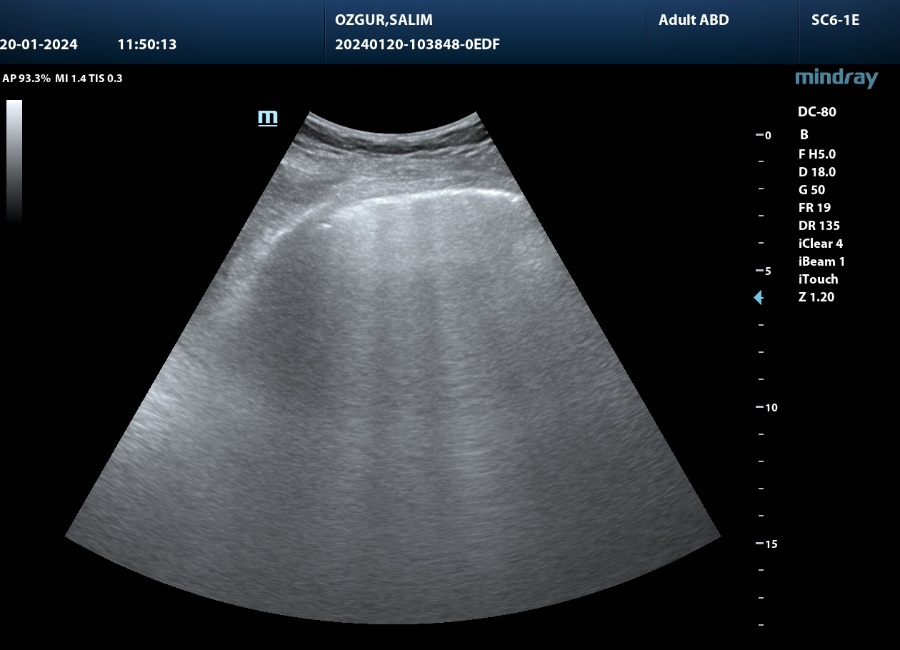 | 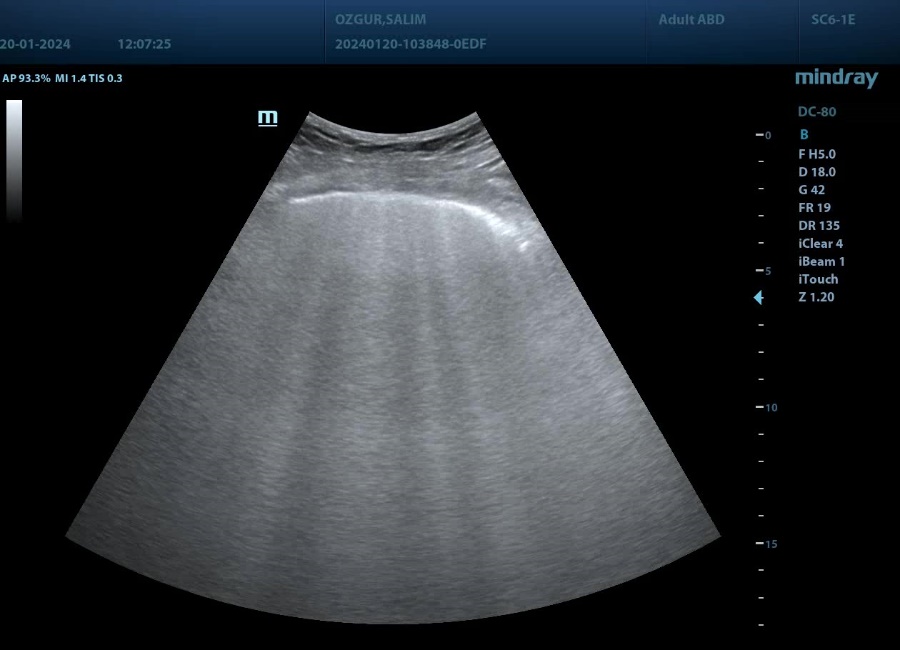 |
| **4** | 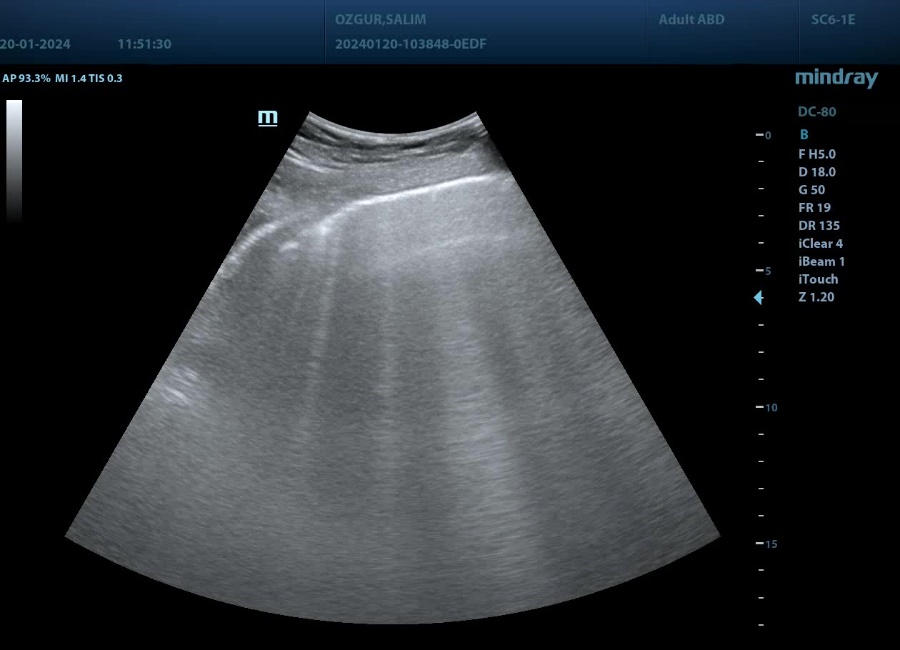 | 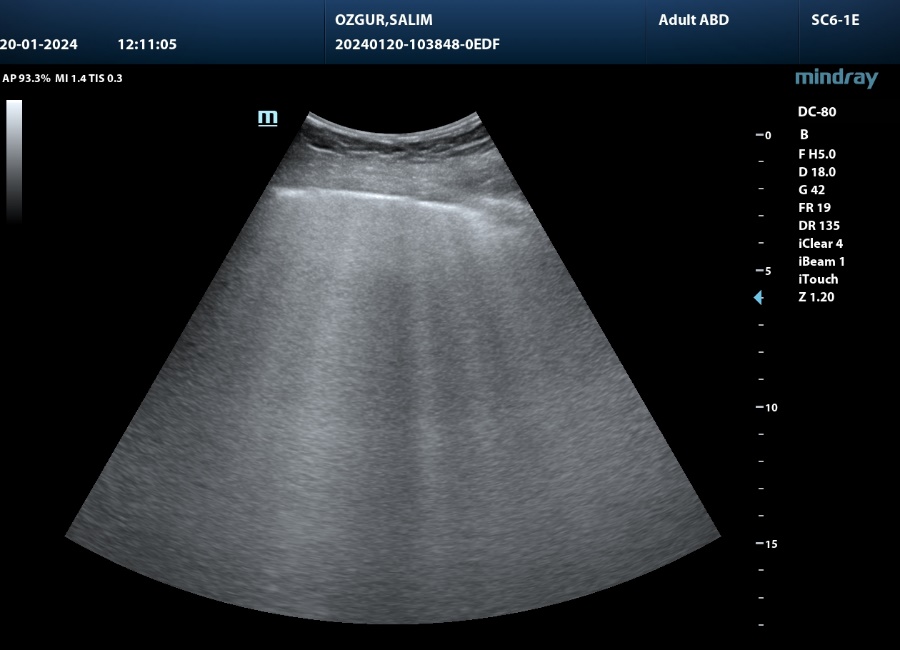 |
| **5** | 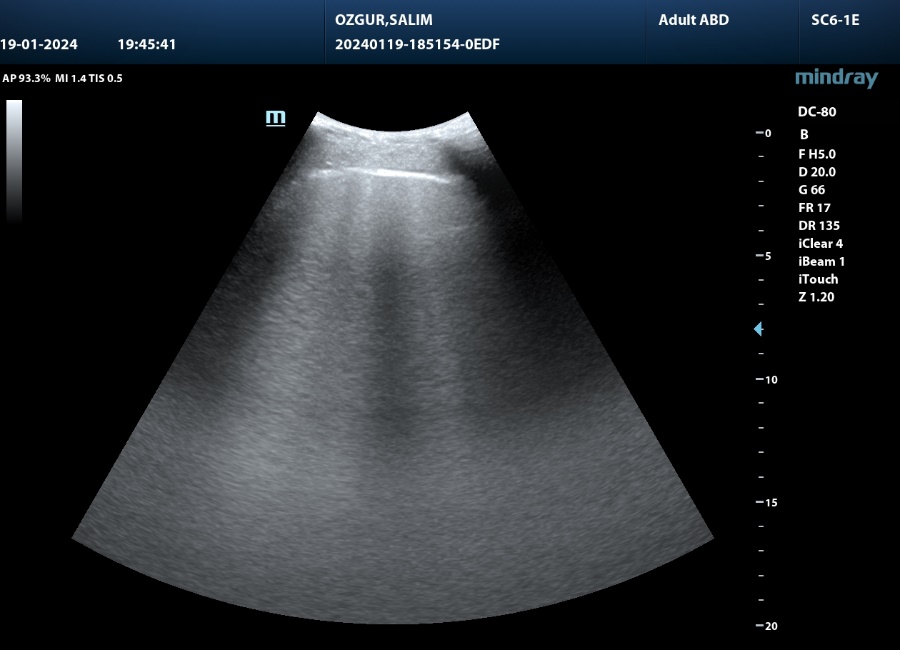 | 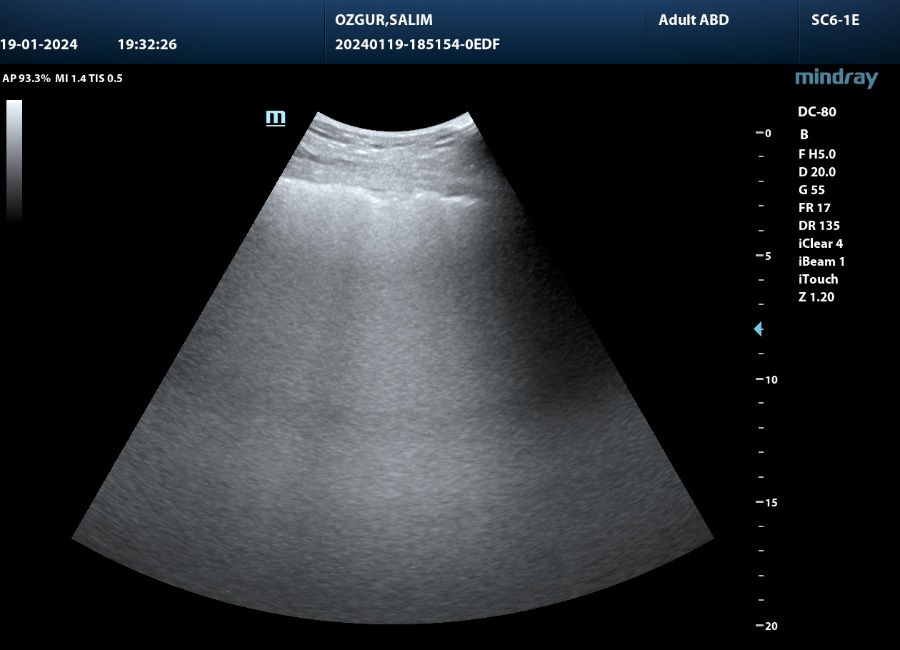 |
| **6** | 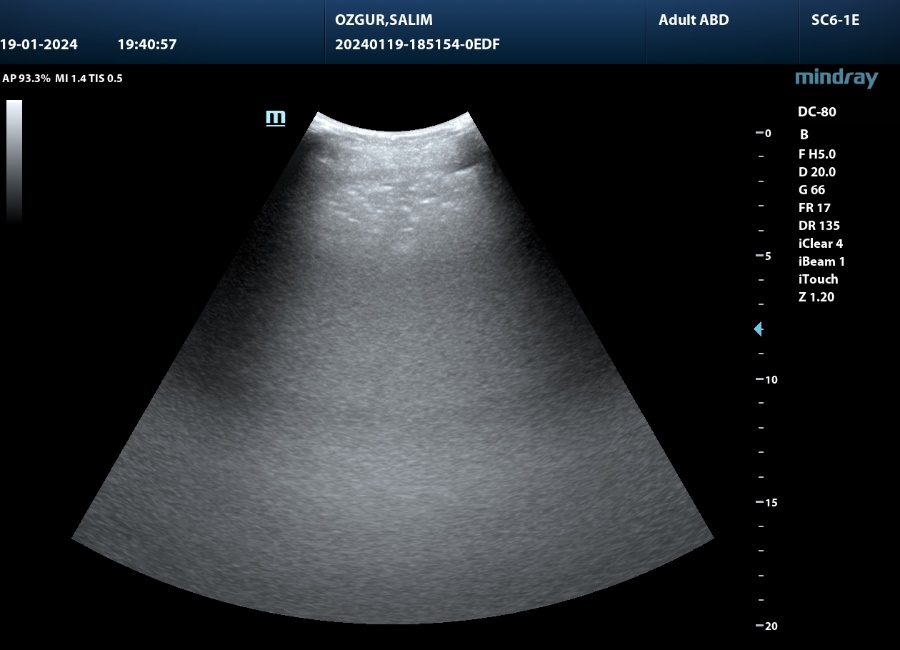 | 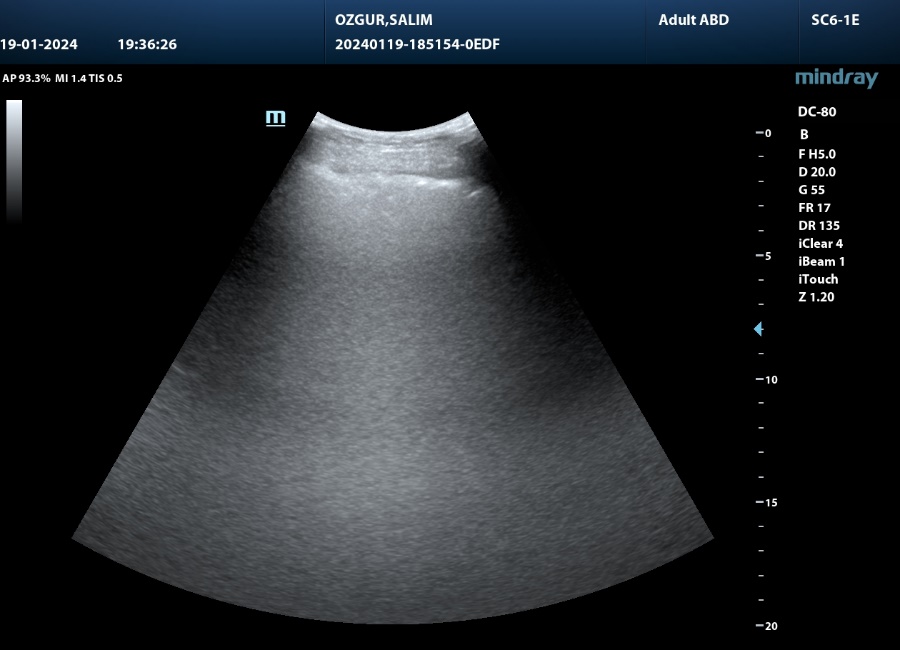 |

**REVERSE TRENDELENBURG POSITION**

| **REGION** | **RIGHT LUNG** | **LEFT LUNG** |
| --- | --- | --- |
| **1** | 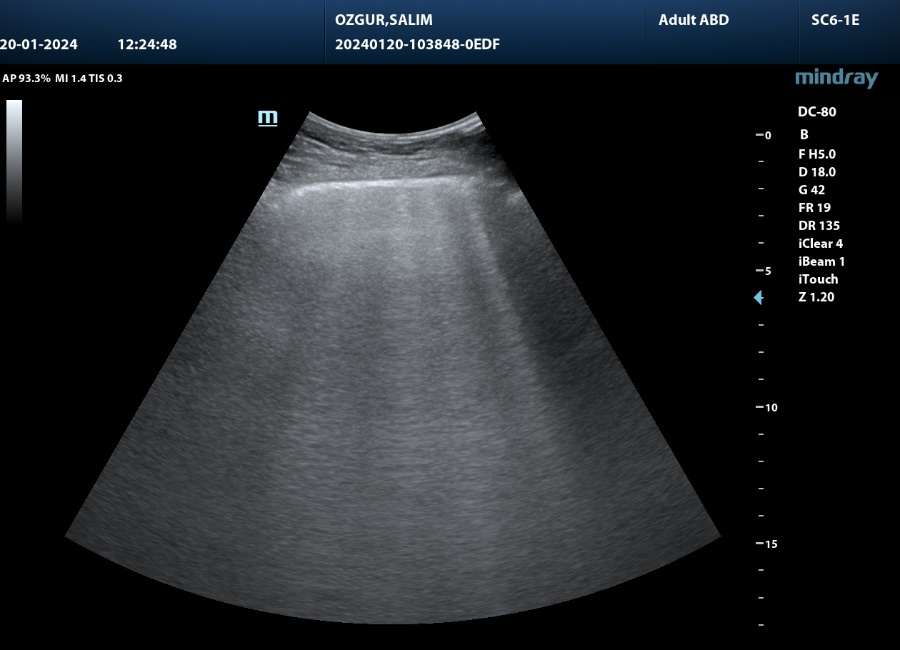 | 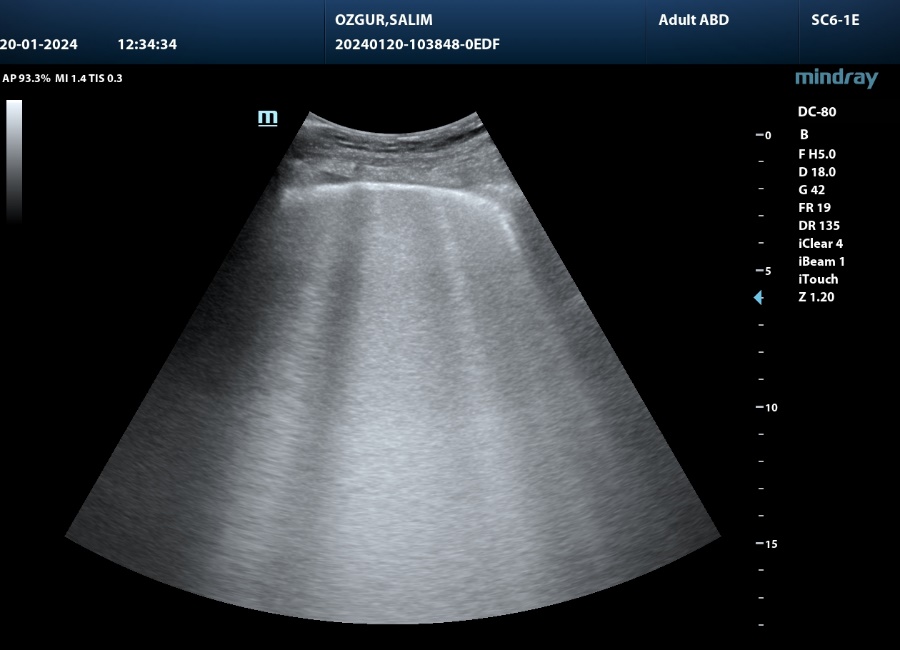 |
| **2** | 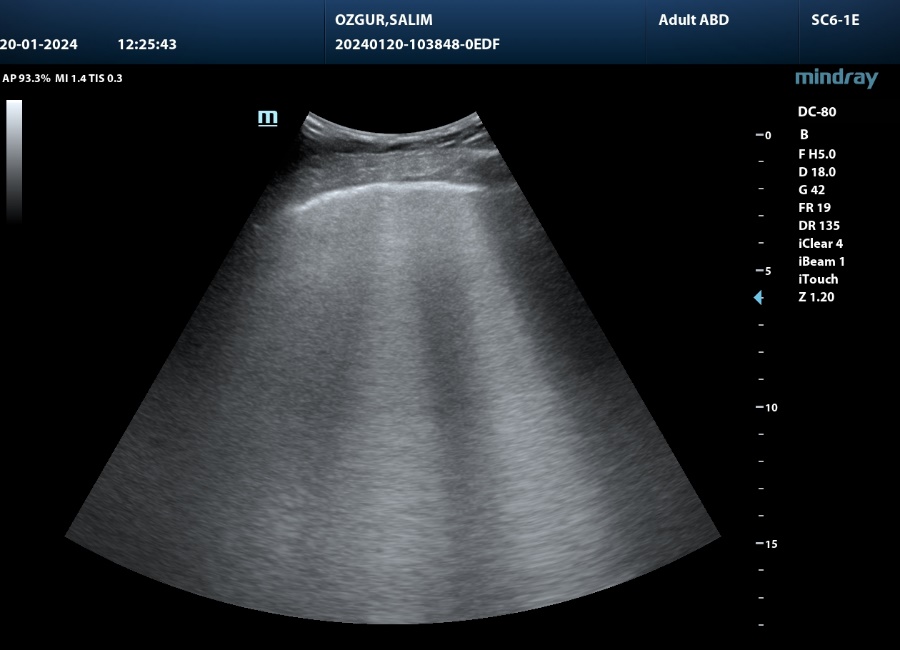 | 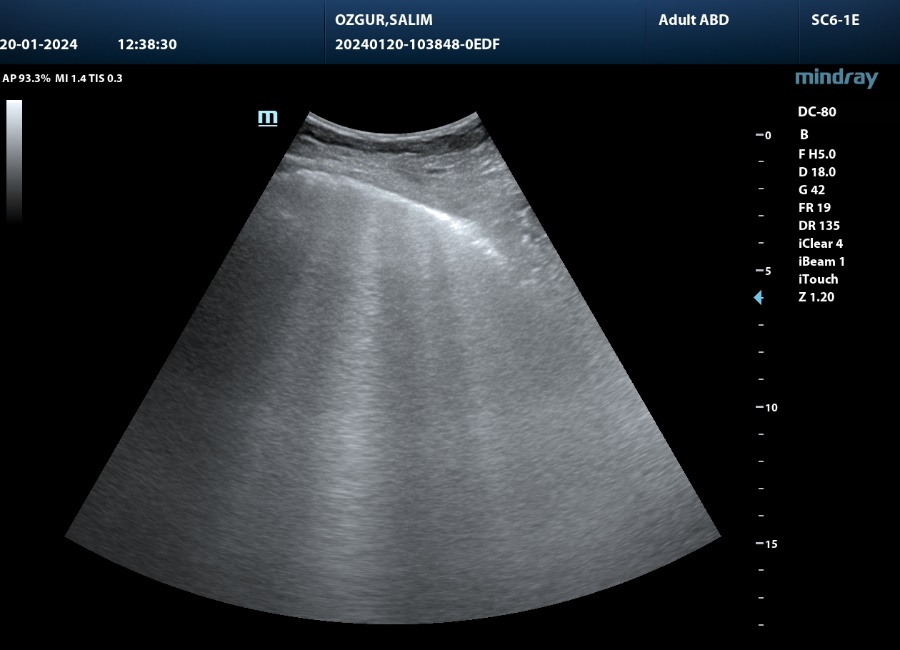 |
| **3** | 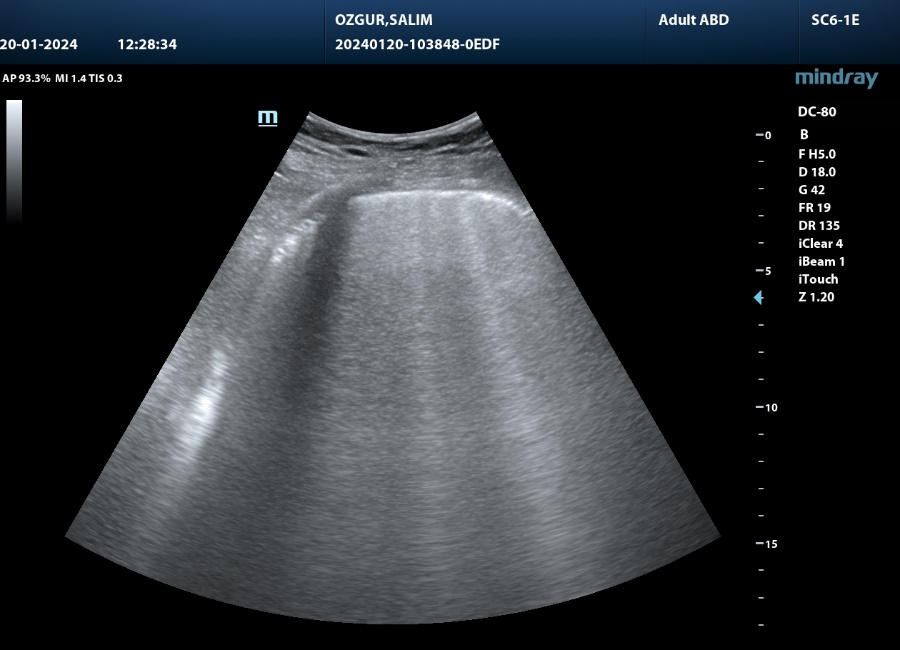 | 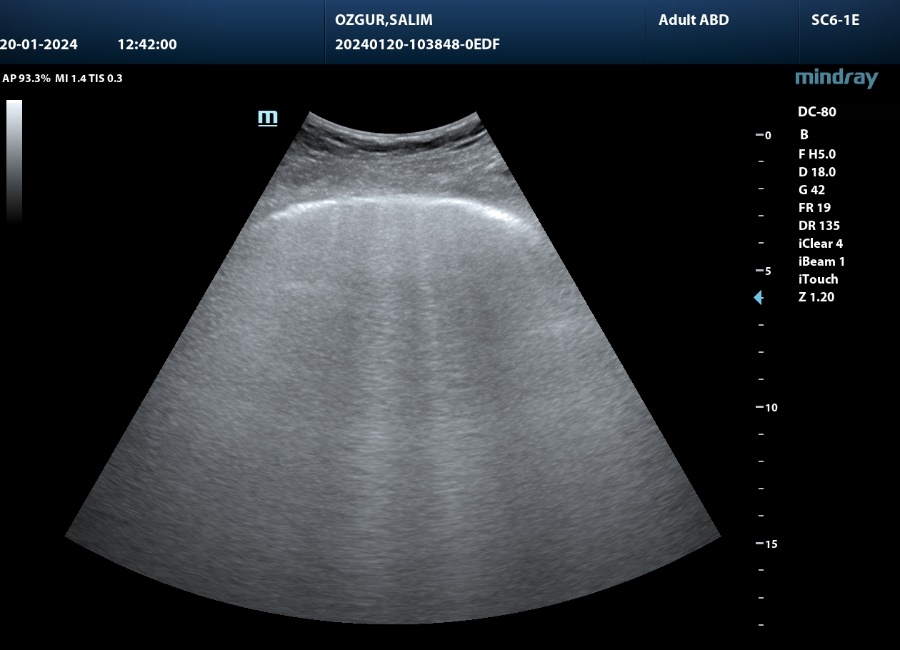 |
| **4** | 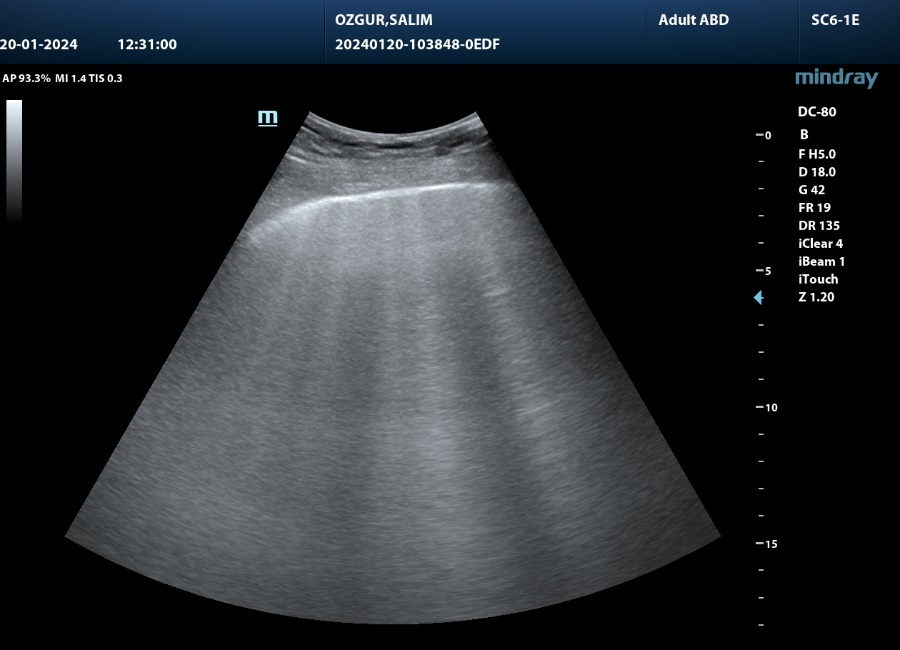 | 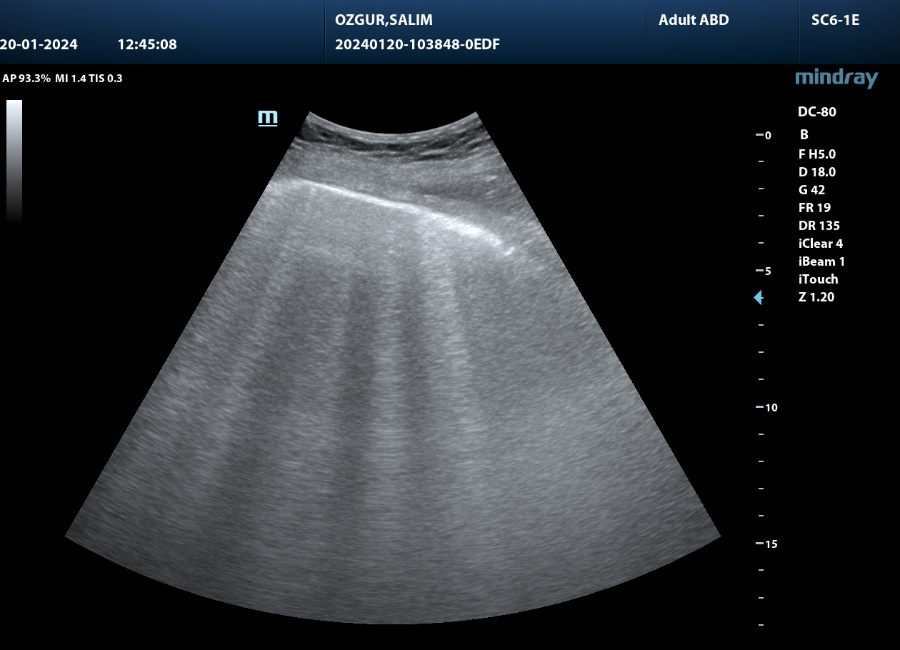 |
| **5** | 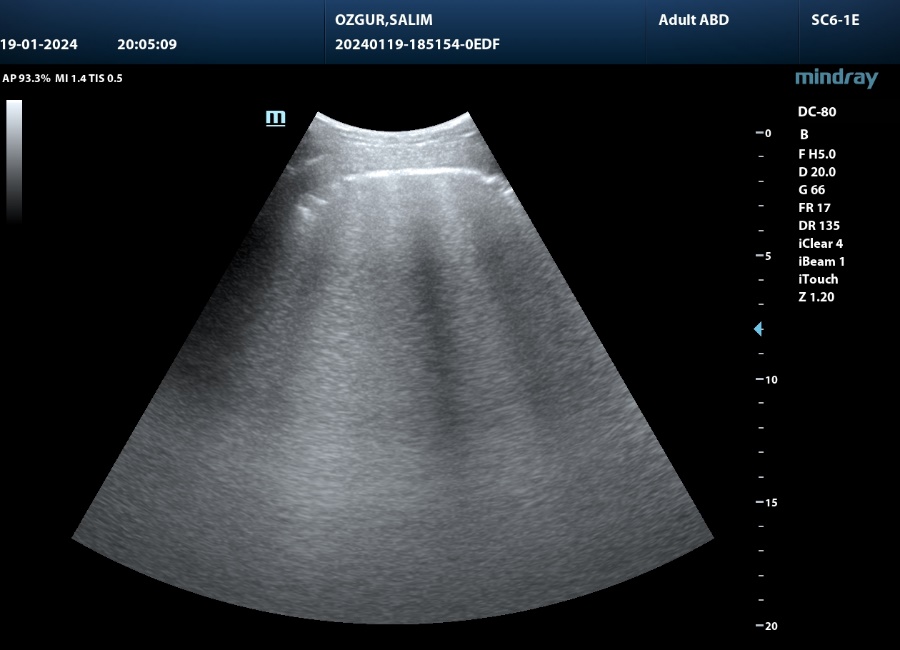 | 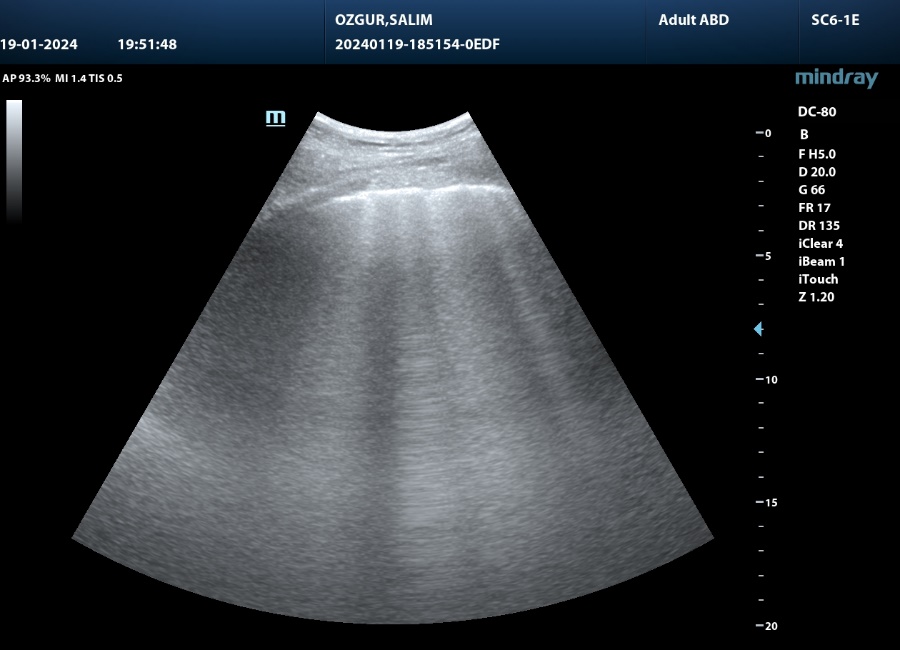 |
| **6** | 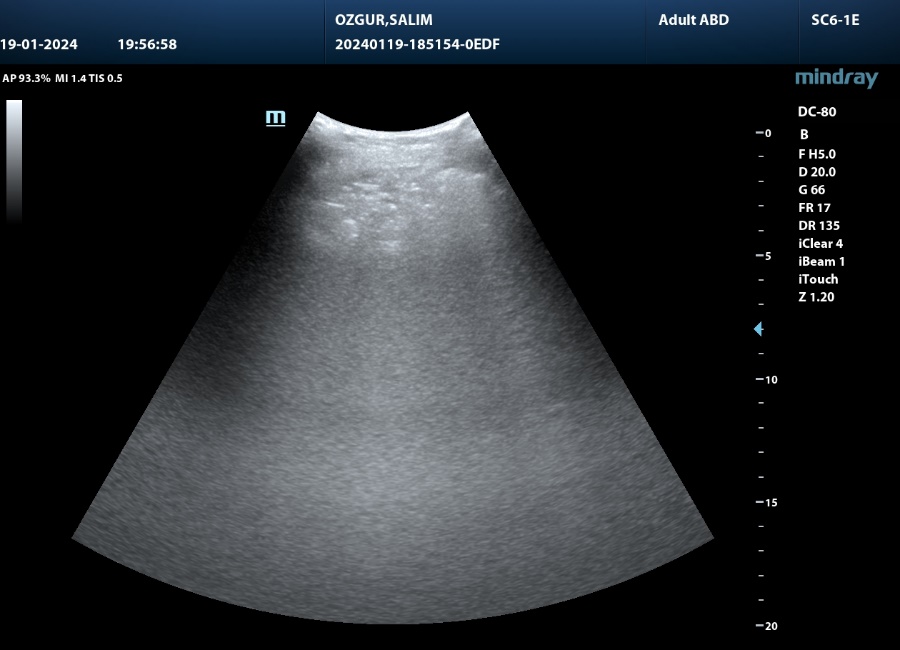 | 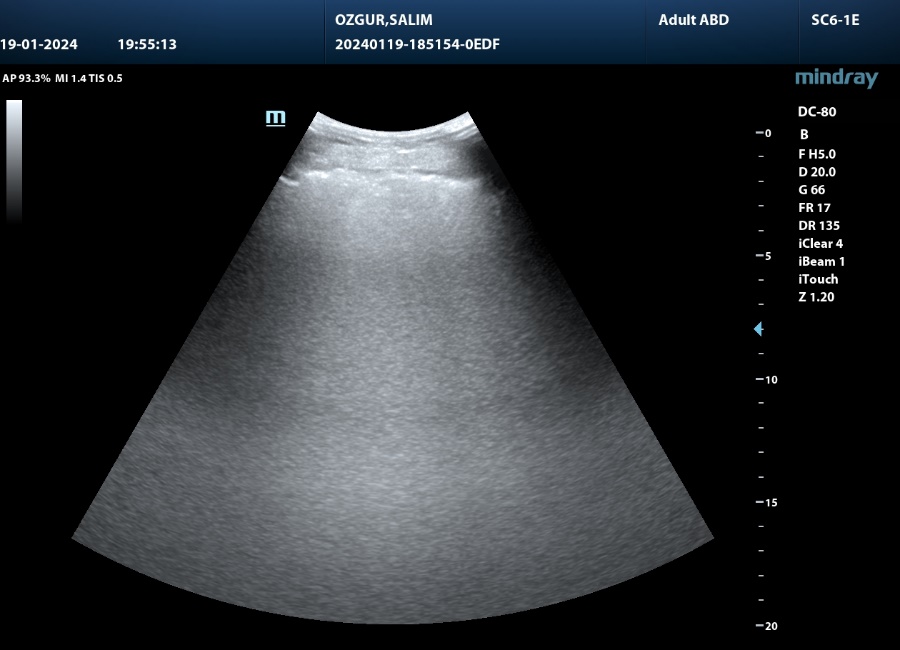 |
