# Supplementary material for: Point‐of‐care Lung ultrasound assessment of positional changes in COVID‐19 ARDS in intensive care: A case report and review of the literature
Source: Physiol Rep. 2025 Aug 22;13(16):e70484. doi: 10.14814/phy2.70484 (PMC12371258; doi:10.14814/phy2.70484)
Supplement: Supplementary file 3 — Table S1. [file PHY2-13-e70484-s003.docx]

**Supplementary Table 1.** Ventilator parameters and duration of positioning prior to lung ultrasound assessment.

| Position | Ventilator Mode | Duration maintained before LUS (minutes) | PEEP (cmH₂O) | FiO₂ (%) | Tidal Volume (ml/kg) | Compliance (mL/cmH₂O) |
| --- | --- | --- | --- | --- | --- | --- |
| Trendelenburg | PRVC | 30 | 10 | 60 | 6 | 37 |
| Flat | PRVC | 30 | 10 | 60 | 6 | 37 |
| Reverse Trendelenburg | PRVC | 30 | 10 | 60 | 6 | 38 |

All measurements were taken during volume control (VC) mode of mechanical ventilation. Lung ultrasound was performed after the patient had remained in each position for 30 minutes. Compliance was calculated based on ventilator-derived values. PEEP: positive end-expiratory pressure; FiO₂: fraction of inspired oxygen.
